# Simulation dataset of thermal and epithermal neutron self-shielding correction factors for 186W(n,γ)187W reaction rate experiments using tungsten foil targets

**DOI:** 10.1016/j.dib.2023.109937

**Published:** 2023-12-09

**Authors:** Trinh T. Tu Anh, Pham N. Son, Chau T. Tai, Truong P. Dung, Trinh V. Cuong, Phan B. Quoc Hieu, Cao D. Vu

**Affiliations:** a Dalat University, 01-Phu Dong Thien Vuong, Dalat, Vietnam; b Dalat Nuclear Research Institute, 01-Nguyen Tu Luc, Dalat, Vietnam; c Faculty of Physics and Engineering Physics, University of Science, Vietnam National University, Ho Chi Minh City, Vietnam

**Keywords:** Neutron cross-section, Neutron self-shielding, Monte-Carlo simulation, Tungsten foil, G_th_, G_epi_

## Abstract

In the experiments of neutron interaction with research samples, the incident neutron energy spectrum, distribution inside the irradiating sample volume, is affected by the unexpected neutron self-shielding effects. The nature of these effects is due to the formation and thickness of the irradiating sample, which significantly causes neutron self-absorption and multiple scattering inside the sample volume. The datasets presented in this article showed the thermal (G_th_) and epithermal (G_epi_) neutron self-shielding correction factors for the ^186^W(n,γ)^187^W neutron capture reaction rate in irradiating tungsten (W) foil samples with different thicknesses. The simulations were performed for three models of surface neutron source's geometries and relative orientations of the irradiating foil samples of isotropic cylinder surface neutron source with foil sample along to the center line, isotropic cylinder neutron source with foil sample flat to the center line, and isotropic spherical neutron source with foil sample placed at the center point. The range of sample thicknesses was from 10 µm to 2.5 mm. The uncertainties for each data point are also reported in the data table, making it more convenient for reuse in related experiments or evaluations.

Specifications Table

**Table:** 

Subject	Physical sciences
Specific subject area	Computational modelling and simulation of correction factors in areas of neutron-induced reaction experiments
Data format	Raw, Analyzed
Type of data	Table and Figure
Data collection	The data were collected from a series of Monte Carlo simulations using the well-known MCNP5 code. The simulations were performed according to three geometry models of neutron source and foil sample, which can be applied to the commonly available neutron irradiation facilities: - Isotropic cylindrical surface neutron source with foil sample placed along the centerline. - Isotropic cylindrical surface neutron source with foil sample placed flat to the centerline. - Isotropic spherical surface neutron source with foil sample placed at the center point. For every simulation run, the neutron radiative reaction rate of ^186^W(n,γ)^187^W reaction occurring inside the natural tungsten (W) metallic foil sample was recorded and obtained from the output file. After that, the self-shielding correction factor was calculated as the ratio of the reaction rate to the corresponding infinite diluted sample reaction rate.
Data source location	Institution: Dalat Nuclear Research Institute Country: Vietnam
Data accessibility	Repository name: Mendeley Data Data identification number: 10.17632/rghrx5dtv4.1 Direct URL to data: https://data.mendeley.com/datasets/rghrx5dtv4/1
Related research article	Trinh T. Tu Anh, Pham N. Son, Nguyen B. Thuy and Cao D. Vu. Thermal neutron capture cross-section and resonance integral measurements of 186W(n,γ)187 W reaction using the thermal column neutron source at the Dalat research reactor, Annals of Nuclear Energy, Volume 194 (2023) 110131 [1]

## 1 Value of the Data

• These datasets are useful in providing the thermal and epithermal neutron self-shielding correction factors for the experiments related to the nuclear reaction 186W(n,γ)187 W using a natural metallic tungsten foil sample.
• The uncertainty information of each data point may be helpful to the users in evaluating the partial propagation uncertainty, which is difficult to determine by other methods or takes a long time for calculation.
• These data provide researchers in this field with supporting knowledge of design and optimal selection of a foil sample thickness for a neutron research irradiation.

## 2 Background

The correction, normalization, or calibration factors are indispensable as supporting data in experimental research. The neutron self-shielding correction is a specific case in the research implementations based on the interaction of neutrons with nuclei, such as measurements of neutron-induced reaction cross-sections, resonance integral, neutron spectrum parameters, and activation analysis. The use of this kind of correction value in the experimental determinations of thermal capture cross-section and resonance integral has been discussed in the literature, and it is briefly described in the related article [1] as follows.

The (n,γ) total reaction rate (R_total_), the thermal neutron reaction rate (R_th_), and epithermal neutron reaction rate (R_epi_) components of a target nucleus in the irradiating sample without Cd-cover, during irradiation in a neutron spectrum ϕ(E), which dominates by thermal with Westcott g-factor and 1/E^1+α^ epithermal neutrons with epithermal α-shape parameter are defined as:

(1) $$R_{th}=G_{th}\phi_{th}g\sigma_{0}$$

(2) $$R_{epi}=G_{epi}\emptyset_{epi}I_{0}\left(\alpha \right)$$

(3) $$R_{total}=G_{th}\phi_{th}g\sigma_{0}+G_{epi}\emptyset_{epi}I_{0}\left(\alpha \right),$$

where G_th_ and G_epi_ are the neutron self-shielding correction factors that can be found suitable values from these datasets [2] for the case of ^186^W(n,γ)^187^W reaction; σ_0_ and I_0_(α) are thermal neutron capture cross-section and resonance integral.

## 3 Data Description

The dataset file (Gth_Gepi_W186_foil_along.txt) contains the simulated values of thermal (G_th_) and epithermal (G_epi_) neutron correction factors for the ^186^W(n,γ)^187^W nuclear reaction in different thicknesses of natural metallic tungsten (W) foil samples [2]. For each simulation run, the foil sample was placed along inside an isotropic cylindrical surface neutron source model.

The dataset file (Gth_Gepi_W186_foil_flat.txt) contains the simulated values of thermal (G_th_) and epithermal (G_epi_) neutron correction factors for the ^186^W(n,γ)^187^W nuclear reaction in different thicknesses of natural metallic tungsten (W) foil samples [2]. For each simulation run, the foil sample was placed flat inside an isotropic cylindrical surface neutron source model.

The dataset file (Gth_Gepi_W186_foil_isotropic.txt) contains the simulated values of thermal (G_th_) and epithermal (G_epi_) neutron correction factors for the ^186^W(n,γ)^187^W nuclear reaction in different thicknesses of natural metallic tungsten (W) foil samples [2]. For each simulation run, the foil sample was placed at the center of a spherical surface neutron source model.

**Fig. 1:**
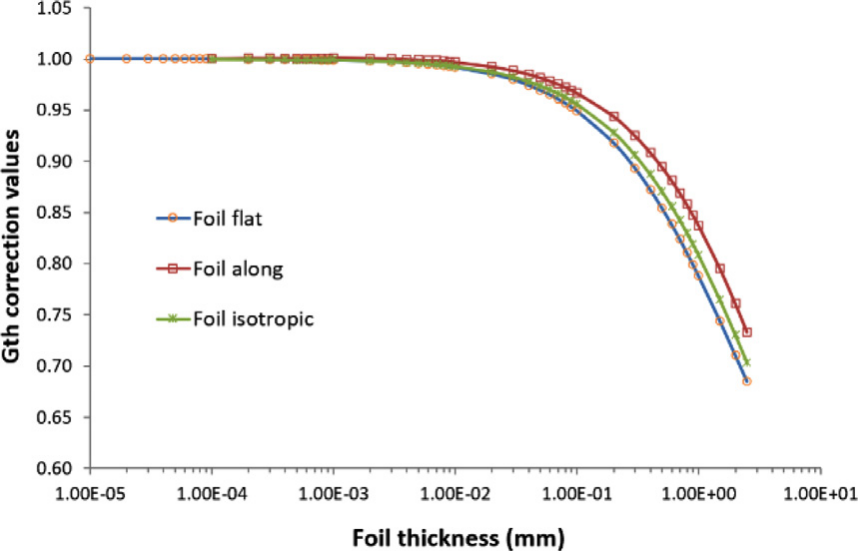
Thermal neutron self-shielding correction factors (G_th_) for ^186^W(n,γ)^187^W reaction in metallic tungsten (W) foil with different thickness.

**Fig. 2:**
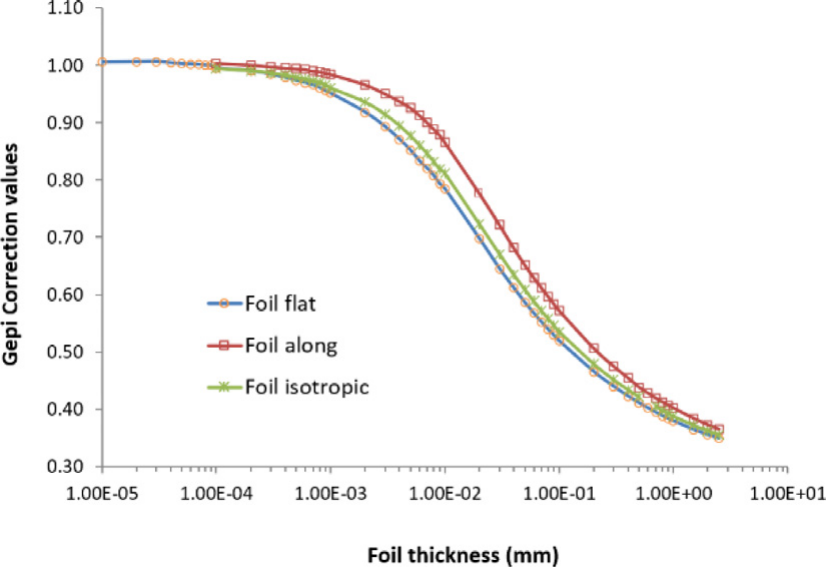
Epithermal neutron self-shielding correction factors (Gepi) for ^186^W(n,γ)^187^W reaction in metallic tungsten (W) foil with different thickness.

The data structure of the above three data files is presented briefly in Table 1, and the datasets are visualized in Figs. 1 and 2.

**Table 1:** Briefly describes information and data structure of the repository data files.

File name:	Gth_Gepi_W186_foil_along.txt				
Reaction: Sample: Experiment:	^186^W(n,γ)^187^W Natural tungsten (W) metallic foil Foil sample placed along inside an isotropic cylindrical surface neutron source				
Order #	Foil thickness (mm)	G_th_	Uncertainty (%)	G_epi_	Uncertainty (%)
1	2.50E+00	7.345E−01	0.06	3.650E−01	0.41
2	2.00E+00	7.627E−01	0.06	3.729E−01	0.42
3	1.50E+00	7.964E−01	0.06	3.842E−01	0.43
4	1.00E+00	8.384E−01	0.06	4.021E−01	0.45
5	9.00E−01	8.482E−01	0.06	4.071E−01	0.46
…	…	…	…	…	…
49	1.00E−05	1.000E+00	0.09	1.000E+00	0.55

File name:	Gth_Gepi_W186_foil_flat.txt				
Reaction: Sample: Experiment:	^186^W(n,γ)^187^W Natural tungsten (W) metallic foil Foil sample placed flat inside an isotropic cylindrical surface neutron source				

Order #	Foil thickness (mm)	G_th_	Uncertainty (%)	G_epi_	Uncertainty (%)

1	2.50E+00	6.851E−01	0.07	3.491E−01	0.21
2	2.00E+00	7.109E−01	0.05	3.555E−01	0.21
3	1.50E+00	7.440E−01	0.07	3.646E−01	0.21
4	1.00E+00	7.881E−01	0.07	3.797E−01	0.22
5	9.00E−01	7.992E−01	0.07	3.840E−01	0.22
…	…	…	…	…	…
49	1.00E−05	1.000E+00	0.11	1.000E+00	0.30

File name:	Gth_Gepi_W186_foil_isotropic.txt				
Reaction: Sample: Experiment:	^186^W(n,γ)^187^W Natural tungsten (W) metallic foil Foil sample placed along inside an isotropic spherical surface neutron source				

Order #	Foil thickness (mm)	G_th_	Uncertainty (%)	G_epi_	Uncertainty (%)

1	2.50E+00	7.039E−01	0.01	3.565E−01	0.13
2	2.00E+00	7.309E−01	0.01	3.636E−01	0.13
3	1.50E+00	7.645E−01	0.01	3.735E−01	0.15
4	1.00E+00	8.084E−01	0.01	3.893E−01	0.17
5	9.00E−01	8.190E−01	0.01	3.943E−01	0.11
…	…	…	…	…	…
49	1.00E−05	1.000E+00	0.02	1.000E+00	0.13

## 4 Experimental Design, Materials and Methods

**Fig. 3:**
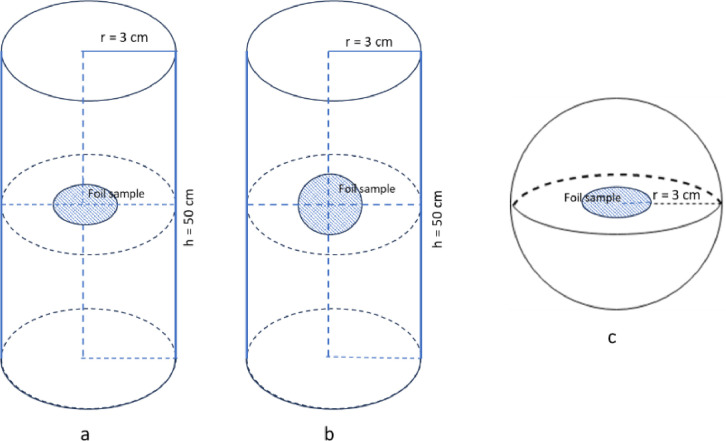
Mechanical models of foil sample irradiations in a neutron field facility: (a) foil placed along a cylindrical neutron irradiation tube, (b) foil placed flat in a cylindrical neutron irradiation tube, and (c) foil placed isotropically in a spherical surface neutron irradiator.

In these simulation works, the Maxwellian and the 1/E spectra of the incident neutron energy distributions were applied for the cases of the thermal (G_th_) and epithermal (G_epi_) neutron self-shielding correction factors, respectively. The Maxwellian distribution of thermal neutrons has an energy region from 1.0 meV to 0.55 eV and the average energy peak at 0.0253 eV. The 1/E distribution for epithermal neutrons has an energy range from 0.55 eV to 200 keV. The MCNP5 code [3], a well-known Monte–Carlo program for the simulation of particle interaction with material, was used in the self-shielding correction factors calculations. The irradiating target samples were metallic tungsten (W) foils with different thicknesses from 0.01 µm to 2.5 mm. The neutron capture reaction rate R(E) as a function of neutron energy (E) per one atom of the irradiating sample is defined as the expression: R(E) = ϕ(E)σ_nγ_(E), where σ_nγ_(E) is the radiative neutron capture reaction cross-section, and ϕ(E) is the neutron flux spectrum. During a neutron irradiation experiment, the ϕ(E) function may significantly decrease at the thermal and resonance energy peaks due to the absorption and scattering interactions, and in the energy regions just lower than the resonance energies, the neutron distribution may increase due to multiple scattering effects [4]. Therefore, this variance behavior in the neutron spectrum can cause uncertainty in the result of the capture reaction rate because of the modification in the neutron spectrum. It is assumed that for a thickness foil sample that has infinitely diluted mass density, the neutron distribution inside this foil sample is a non-modified spectrum. The neutron self-shielding correction factor for a specific thickness foil sample can be calculated as follows [5].

(4) $$\begin{array}{c} G_{\mathrm{th}}=\frac{\int_{E_{0}}^{E_{\mathrm{Cd}}}\phi \left(E\right){\sigma_{n}}_{\gamma }\left(E\right)\mathrm{dE}}{\int_{E_{0}}^{E_{\mathrm{Cd}}}\phi_{0}\left(E\right)\sigma_{n\gamma }\left(E\right)\mathrm{dE}} \end{array}$$

(5) $$G_{epi}=\frac{\int_{E_{Cd}}^{E_{0.2MeV}}\phi \left(E\right)\sigma_{n\gamma }\left(E\right)dE}{\int_{E_{Cd}}^{E_{0.2MeV}}\phi_{0}\left(E\right)\sigma_{n\gamma }\left(E\right)dE},$$

where ϕ_0_(E) is the original or non-affected neutron spectrum; ϕ(E) represents the affected neutron spectrum inside the irradiating foil sample; E_0_ and E_Cd_ are, respectively, the lower and upper limits of the thermal neutron spectrum, for G_th_: E_0_ = 0.01 meV and E_Cd_ = 0.55 eV (Cd cutoff energy). The non-affected and affected neutron spectrums inside a foil sample volume were simulated using the MCNP5 code [3] with the ENDF/B-VII nuclear data library [6]. The simulation models of the neutron source's geometries and relative orientations of the irradiating W foil samples are presented in Fig. 3, and described in detail in reference [7].

## Limitations

These data sets may not be applicable in the cases of wire samples and collimated neutron beams.

## Ethics statement

The authors have read and follow the ethical requirements for publication in Data in Brief and confirming that the current work does not involve human subjects, animal experiments, or any data collected from social media platforms.

## CRediT authorship contribution statement

**Trinh T. Tu Anh:** Project administration, Conceptualization, Methodology, Visualization, Writing – review & editing. **Pham N. Son:** Methodology, Software, Validation, Formal analysis, Investigation, Data curation, Writing – review & editing. **Chau T. Tai:** Data curation, Software, Investigation. **Truong P. Dung:** Data curation, Software. **Trinh V. Cuong:** Data curation, Software. **Phan B. Quoc Hieu:** Data curation, Software. **Cao D. Vu:** Supervision, Formal analysis, Validation.

## Data Availability

Thermal and epithermal neutron self-shielding correction factors for neutron radiative capture reaction of 186W in tungsten foils (Original data) (Mendeley Data)
